# Making judgments of learning (JOLs) for oneself versus others: A review and proposed model

**DOI:** 10.3758/s13423-025-02816-0

**Published:** 2025-12-15

**Authors:** Yunfeng Wei, Nicholas C. Soderstrom, Michelle L. Meade

**Affiliations:** Department of Psychology, Montana State University, P.O. Box 173440, Bozeman, MT 59717-3440 USA

**Keywords:** Judgments of learning (JOLs), JOLs for others, Metacognition, Cue-utilization

## Abstract

A judgment of learning (JOL) refers to an individual’s evaluation and prediction of their own learning. Converging evidence suggests that making JOLs is an inferential process, with individuals basing their judgments on a variety of cues that may or may not be diagnostic of future performance. Compared to such JOLs for oneself, considerably less is known about making JOLs for *others*, but the existing evidence on this topic also supports an inferential-based process. Importantly, although there appears to be substantial overlap regarding the cues used to inform both types of judgments, there are likely cues utilized when forming JOLs for others that are not used when making JOLs for oneself. In the current article, we (1) review the extant literature on JOLs for oneself to understand the characteristics and underlying mechanisms of such judgments; (2) review existing literature regarding JOLs for others, highlighting the similarities and differences between these judgments and those made for oneself; and (3) propose a new model, adapted from Koriat’s (*Journal of Experimental Psychology: General, 126* (4), 349–370, Koriat, Journal of Experimental Psychology: General 126:349–370, 1997) cue-utilization framework, to describe how JOLs for others are made. Our new model provides a substantial theoretical contribution to the literature that can inform both basic and applied research focusing on JOLs for others.

## Introduction

A judgment of learning (JOL) is a metacognitive judgment in which an individual evaluates the likelihood that he or she will remember information in the future. These prospective judgments can be made for single items (e.g., “What is the likelihood that you will remember this fact on a later test?”) or entire sets of materials (e.g., “What percentage of this passage do you think you will be able to remember on a later test?”). Researchers started their work to investigate JOLs decades ago and have published a myriad of papers about individuals’ judgment of learning (for reviews, see Dunlosky & Metcalfe, 2009; Rhodes, 2016, 2019). However, most previous studies focused on JOLs for oneself instead of JOLs for others; thus, researchers have gained comprehensive knowledge about the former but lack a full understanding of the latter. In daily life, individuals not only make JOLs for themselves but also for others. For example, teachers need to judge students’ learning in order to adjust their instruction to improve students’ academic performance (Thiede et al., 2019). Such JOLs for others are also critical in criminal and civil systems because it is necessary to evaluate how well witnesses remember experienced events (Helm & Growns, 2023). Also, tutors, whether human or artificial intelligence (AI), must make JOLs for students. For example, MetaTutor, a hypermedia environment that helps students self-regulate their learning, involves JOLs and other monitoring processes to assist learners in achieving their learning goals (for a review, see Azevedo et al., 2011). Thus, the current paper aims to fill a gap in the literature by reviewing papers relevant to making JOLs for others and proposing a new model to help explain how such social JOLs are made. To this end, we first review articles focused on JOLs for oneself and their proposed theoretical accounts. Next, we discuss the extant literature on JOLs for others, relating it to the literature on JOLs for oneself, and highlight the similarities and differences between these two types of judgments. We then propose and describe a new model, adapted from Koriat’s (1997) cue-utilization framework, that encompasses the cues individuals use when making inferential judgments about others’ learning. Finally, we delineate future directions for this important line of research.

## Judgments of learning for oneself

### Definition and measures of accuracy

JOLs belong to a family of metacognitive monitoring judgments (for more details, see Dunlosky & Metcalfe, 2009; Nelson & Narens, 1990). These judgments are made during or after acquisition in order to evaluate learning and predict future performance. An individual can make item-by-item JOLs or aggregate JOLs for a set of to-be-learned materials. JOL accuracy can be measured in two ways: calibration (absolute judgment) and resolution (relative judgment; for reviews, see Dunlosky & Metcalfe, 2009; Rhode, 2016, 2019; Soderstrom et al., 2016). Calibration refers to the absolute difference between memory performance and JOLs. For example, a student might predict that they will remember 70% of studied information on a later test, but their final performance on that test is 80%. In terms of calibration, this would indicate that the student is 10% underconfident about their performance. Resolution, on the other hand, refers to the correspondence between JOLs and memory performance on an item-by-item basis and is usually quantified by computing Goodman–Kruskal gamma correlations. Specifically, resolution measures the extent to which individuals make higher JOLs for remembered items and lower JOLs for unremembered items. For example, say a learner is 60% confident that they will remember Item A and 50% confident they will remember Item B. If, on a later test, Item A is remembered and Item B is not, resolution would be high; resolution would be low if Item B was remembered, and Item A was not. Thus, calibration is a relatively general evaluation of learned materials, whereas resolution is concerned with item-by-item evaluations.

In general, the accuracy of JOLs is relatively low because JOLs are susceptible to cues that are not predictive of individuals’ later learning performance (Carpenter et al., 2020a, 2020b; Castel, 2008; de Blume & Londoño, 2022; Hertzog et al., 2003; Kornell & Hausman, 2017; Koriat & Bjork, 2005; Koriat et al., 2002; Rhodes & Castel, 2009; Yang et al., 2018; for reviews, see Dunlosky & Metcalfe, 2009; Dunlosky et al., 2015; Rhodes, 2016, 2019). However, many researchers have found that delayed JOLs – making JOLs after a short delay following acquisition – are more accurate than immediate JOLs (Dunlosky & Nelson, 1992; Foster et al., 2023; Jönsson et al., 2012; Kelemen & Weaver, 1997; Metcalfe & Finn, 2008; Nelson & Dunlosky, 1991; Pyc et al., 2014; Yang et al., 2017; for a review, see Rhodes & Tauber, 2011). For example, Nelson and Dunlosky (1991) found that the mean accuracy (Gamma correlation) of immediate JOLs is +.38, but a short delay between acquisition and making JOLs can increase accuracy to +.90. The well-accepted explanation is that when individuals delay their JOLs, they tend to base their judgments on a more diagnostic cue – namely, retrieval practice (Dunlosky & Nelson, 1992; Metcalfe & Finn, 2008; Pyc et al., 2014; for a review, see Rhodes & Tauber, 2011; cf. Spellman & Bjork, 1992), though other researchers have claimed this may be due to other factors such as attention shifts (Bui et al., 2018), past experience activation (Kelley et al., 2021), or item discrimination (Nelson et al., 2004). The retrieval hypothesis is supported, in part, by the finding that retrospective confidence judgments (i.e., assessing confidence in remembered items) are more accurate than prospective JOLs (e.g., Dougherty et al., 2005, 2018).

### Functions of judgments of learning (JOLs) for oneself

Perhaps the most important function of JOLs is to guide future self-regulated learning (e.g., Händel & Dresel, 2022; Morehead & Dunlosky, 2020; Morehead et al., 2017; Son & Metcalfe, 2000; Son & Sethi, 2006; Thiede, 1999; Toppino & Pagano, 2021; Toppino et al., 2018; Yu et al., 2020; for reviews, see Bjork et al., 2013; Kornell & Finn, 2016). That is, when regulating future study sessions, individuals will take their subjective assessments of their own learning into consideration when adjusting time allocation, selecting information to study, choosing various learning strategies, and so on. If learners’ JOLs are accurate, their restudy decision may be productive, but if their JOLs are not accurate, their restudy decision may lead to minimal gain, or even be counterproductive (Bjork et al., 2013; Dunlosky & Rawson, 2012; Kornell & Bjork, 2008; Kornell & Metcalfe, 2006). In support of the guiding role of JOLs on self-regulated learning, researchers have found that the accuracy of JOLs is positively related to later test performance as measured by calibration (e.g., Dunlosky & Rawson, 2012; Händel et al., 2020; Rawson et al., 2011) and resolution (e.g., Cogliano et al., 2021; Rawson et al., 2011) when learning relatively complex materials. However, it is worth noting that Dunlosky et al. (2021) demonstrated that monitoring can produce memory gains only when restudy can produce substantial improvement and when individuals select their learning strategies appropriately. Thus, accurate JOLs can improve learning performance by optimizing subsequent self-regulated learning activities.

Interestingly – and perhaps surprisingly – the mere act of making JOLs may have a direct influence on some types of learning. For example, making immediate JOLs can enhance associative learning (Halamish & Undorf, 2023; Maxwell & Huff, 2022; Myers et al., 2020; Senkova & Otani, 2021; Soderstrom et al., 2015; for a review, see Double et al., 2018), although it is still unclear how making JOLs might influence other types of learning. Making delayed JOLs may also affect learning itself (see Kimball & Metcalfe, 2003; Spellman & Bjork, 1992). Jönsson et al. (2012), for example, found that making delayed JOLs can enhance learning about as much as testing, or retrieval practice., although it should be noted that other researchers have not found a direct effect of making JOLs on learning (e.g., Dougherty et al., 2018). In addition, there is evidence that making JOLs can potentiate the learning of *new* items (e.g., Kubik et al., 2022; Lee & Ha, 2019). On the whole, the processes involved in the act of making JOLs seem to improve some types of learning, but future research is needed to clarify which types of learning, exactly.

### Mechanism for JOLs for oneself

Two major theoretical accounts have been put forward to describe the basis for making JOLs for oneself: the direct access account and the inferential account (for a review, see Rhodes, 2016). The direct access account argues that individuals have direct access to their memory traces for studied items and base their JOLs on such traces, giving relatively high JOLs to items with stronger memory traces and relatively low JOLs to items with weaker traces. This account has been supported by recent neuroimaging findings showing that brain regions activated during retrieval are also activated when making JOLs (e.g., Do Lam et al., 2012; Hu et al., 2017; Kelley et al., 2020, 2021). However, most of the evidence has supported the inferential account, according to which learners base their judgments on a variety of available cues, some of which may be diagnostic of future performance and some of which may not.

**Fig. 1:**
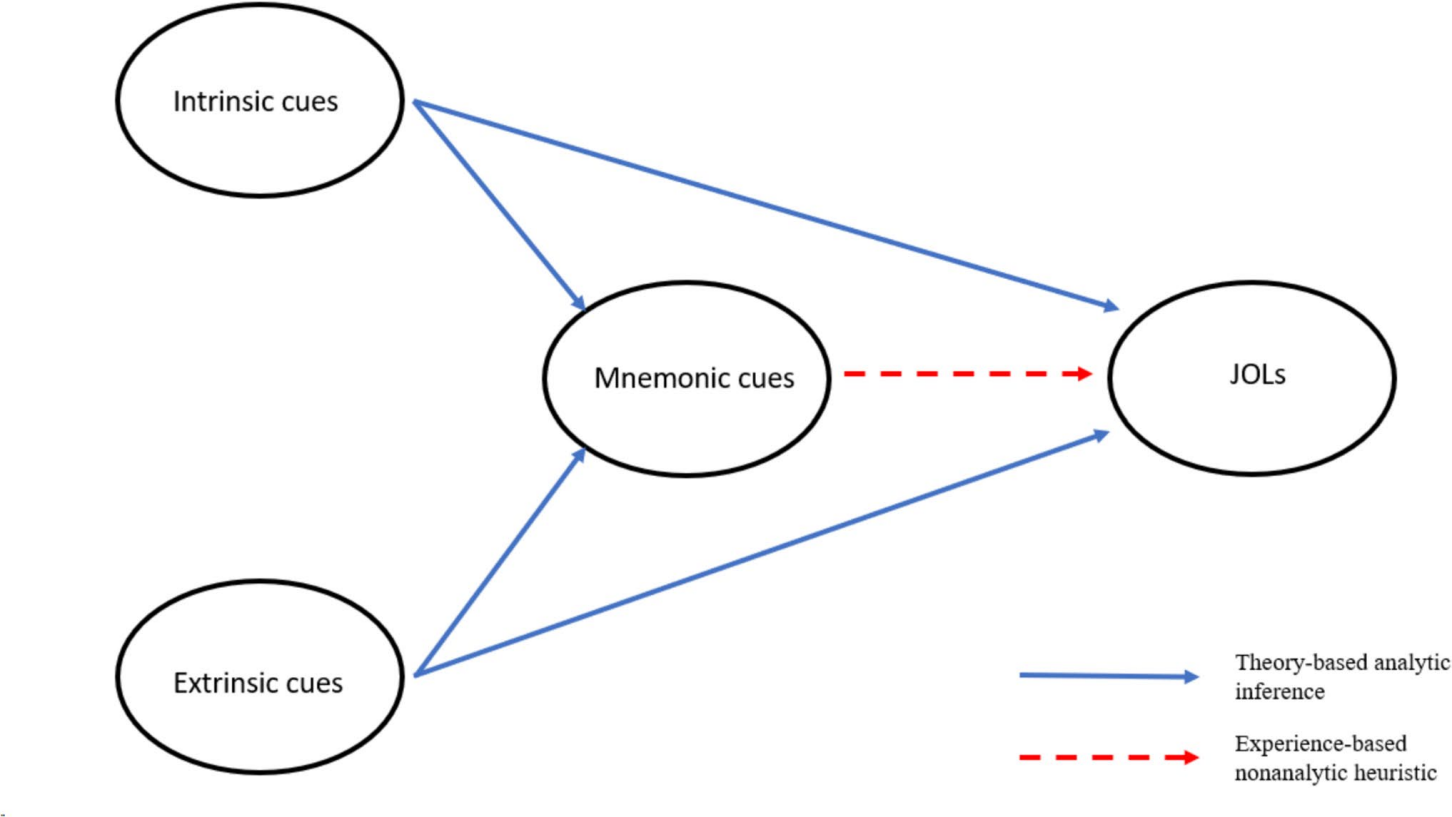
Direct and indirect effects of cues on judgments of learning (JOLs). Model adapted from Koriat (1997)

Koriat’s (1997) cue utilization model has been the most prominent inferential account of JOLs, the basics of which are shown in Fig. 1. According to Koriat, three major categories of cues can be used when making JOLs: intrinsic cues, extrinsic cues, and mnemonic cues. Both intrinsic and extrinsic cues lead to theory-based analytical inferences, meaning that individuals have theories about how these cues will affect their learning and thus incorporate those beliefs into their JOLs when those cues are used. Mnemonic cues, on the other hand, result in experienced-based non-analytic inferences. Here, idiosyncratic experiences, not a priori beliefs, influence the formation of JOLs. We now describe in more detail the differences between intrinsic, extrinsic, and mnemonic cues, reviewing the relevant literature as we do so.

#### Intrinsic cues

Intrinsic cues refer to the characteristics of the materials that individuals intend to learn, including associative strength, emotionality, font size, and so on. Early research indicated that intrinsic cues commonly track the difficulty of the to-be-learned materials (e.g., Lovelace, 1984; Lovelace & Marsh, 1985; Rabinowitz et al., 1982; Underwood, 1966), and thus individuals should be able to make relatively accurate JOLs for themselves if they have access to intrinsic cues. However, subsequent work has shown that some intrinsic cues are not necessarily diagnostic of future performance and, therefore, can lead to impaired metacognitive accuracy. Some intrinsic cues, such as associative relatedness (e.g., Koriat, 1997; Mueller et al., 2012; Underwood, 1966), emotionality (e.g., Zimmerman & Kelley, 2010), and imagery value (e.g., Begg et al., 1989; Groninger, 1979), influence memory and JOLs in the same way. If individuals make JOLs based on these cues, the accuracy of JOLs will be relatively high. However, this is not always the case, as some intrinsic cues have been found to influence JOLs independent of memory performance, and vice versa, leading to relatively low accuracy. For example, cues such as font size (e.g., Rhodes & Castel, 2008), presentation clarity (e.g., Carpenter et al., 2020a, 2020b; Yue et al., 2012), and auditory intensity (e.g., Rhodes & Castel, 2009; Soderstrom & Rhodes, 2014) lead to changes in JOLs but have negligible effects on memory. As a result, individuals will show metacognitive illusions such that their JOLs fluctuate in accordance with these cues, whereas their later memory performance does not (e.g., learners JOLs indicate that larger words will be better remembered than smaller words, but later memory performance does not vary as a function of font size; for an exception, see Maxwell et al., 2022). The inverse is also true – namely, certain cues can impact learning but have little impact (or even the opposite effect) on JOLs. For example, disfluency during learning is often conducive to learning, but individuals rarely detect this beneficial effect when making JOLs. Hard-to-read font (Diemand-Yauman, Oppenheimer & Vaughan, 2010) and inverted words (Sungkhasettee et al., 2011) have produced memorial benefits, yet JOLs do not track such benefits, leading to another kind of metacognitive illusion.

To summarize, intrinsic cues are inherent to the materials being studied, lead to theory-based analytical inferences, and can have various effects on JOLs and memory performance. Some intrinsic cues affect JOLs and memory in similar ways; others can lead to metacognitive illusions where the cues affect either JOLs or memory performance, but not both. Thus, intrinsic cues are only diagnostic when they influence memory and JOLs in similar ways.

#### Extrinsic cues

According to Koriat (1997), extrinsic cues refer to: (1) the conditions under which individuals learn materials, such as the number of repetitions (e.g., Lovelace, 1984; Zechmeister & Shaughnessy, 1980), presentation time (e.g., Koriat et al., 2009; Mazzoni et al., 1990), and distributed versus massed repetition (e.g., Dunlosky & Nelson, 1994; Zechmeister & Shaughnessy, 1980); and (2) the learning strategies applied by individuals during acquisition, including level of processing (Rabinowitz et al., 1982; Shaw & Craik, 1989) and the use of imagery (Begg et al., 1989, 1991; Dunlosky & Nelson, 1994). Again, extrinsic cues (like intrinsic cues) lead to theory-based analytic inferences, meaning that people apply their theories about how these cues *should* affect their learning when making JOLs.

Koriat (1997) provided evidence that individuals tend to discount extrinsic cues when making JOLs. Participants experienced multiple study-test cycles, and it was found that participants’ learning performance increased from 55 to 85%, whereas their JOLs only increased from 50 to 65% over the cycles. That is, individuals had difficulty discerning their improvement after repeated practice, leading to under-confidence about their performance. This specific pattern of relatively low calibration accuracy over multiple study-test cycles is known as the under-confidence-with-practice (UWP) effect (see also England & Serra, 2012; Koriat et al., 2002, 2006). Other studies have supported this discounting effect by showing that individuals demonstrate a *stability bias*, misbelieving that their memory performance will remain consistent regardless of the retention interval (the extrinsic cue) between acquisition and the final memory test (for reviews, see Bjork et al., 2013; Kornell, 2011; Soderstrom & Bjork, 2015). In contrast to calibration, resolution is often relatively high when extrinsic cues are noticed and used. For example, Koriat (1997) found that both memory and JOLs increased after repetition, which enhanced resolution (see also Undorf et al., 2018). Likewise, Jang and Nelson (2005) reported relatively high resolution in experiments in which individuals made higher JOLs and performed better with longer presentation times and repeated studying. High relative accuracy was also found by Dunlosky and Nelson (1994), who had participants engage in either interactive imagery or rote rehearsal during acquisition.

To summarize, extrinsic cues refer to the learning conditions and strategies present during acquisition. Making JOLs based on extrinsic cues can sometimes impair calibration, usually causing under-confidence or over-confidence, but resolution of such JOLs is often relatively high. Similar to intrinsic cues, extrinsic cues are theory-based analytic cues.

#### Mnemonic cues

Mnemonic cues refer to idiosyncratic indicators that can be used to predict future memory performance, including accessibility of relevant information (e.g., Dunlosky & Nelson, 1992; Koriat, 1993; Morris, 1990), difficulty level in retrieval (e.g., Kelley & Lindsay, 1993; Koriat, 1993; Mazzoni & Nelson, 1995), familiarity with cues (e.g., Metcalfe et al., 1993; Reder & Ritter, 1992), retrieval fluency (Begg et al., 1989; Benjamin & Bjork, 1996), prior memory performance (e.g., Gardiner et al., 1977; King et al., 1980), and domain-specific knowledge (e.g., Glenberg & Epstein, 1987; Glenberg et al., 1987; Serra & Shanks, 2023; Shanks & Serra, 2014), and so on.

Koriat (1997) reported that individuals gradually shift their basis for making JOLs from intrinsic cues to mnemonic cues as practice increases, and when JOLs are based on mnemonic cues, accuracy of JOLs tends to be high. These findings are consistent with previous studies showing that individuals rely heavily on intrinsic cues when making immediate JOLs, but they base their JOLs more on mnemonic cues and less on intrinsic cues (Carroll et al., 1997). Making JOLs based on intrinsic and extrinsic cues is thought to be a theory-based, analytic process, whereas mnemonic cues provide individuals with *experience*-based heuristics to predict their future performance (Jacoby & Brooks, 1984; Koriat, 1994). Koriat also pointed out that mnemonic cues are typically highly diagnostic of future performance. The findings of Lovelace (1994) supported this view by showing that even though there is commonality in individuals’ JOLs, their JOLs can also be accounted for by idiosyncratic and privileged components. Thus, mnemonic cues are diagnostic and predictive of future performance (Koriat, 1993).

#### Summarization of cue-utilization theory

Koriat (1997) delineated the effects of intrinsic, extrinsic, and mnemonic cues on JOLs. This model shows that intrinsic and extrinsic cues are theory-based, meaning that individuals rely on their pre-existing theoretical notions about how such cues should affect their future performance. Mnemonic cues, on the other hand, are idiosyncratic and experience-based cues that are unique to each learner. It is worth noting that intrinsic and extrinsic cues can influence JOLs in both direct and indirect ways (through mnemonic cues), and as a result, it is hard to discern the pure effect of some intrinsic and extrinsic cues. For instance, better resolution of JOLs after repeated learning can be attributed to an inference about repetition of learning (extrinsic, theory-based cue) or prior learning experiences (mnemonic, experience-based cue). Also, individuals can take multiple cues into account when making JOLs. For example, Undorf et al. (2018) revealed that individuals can use multiple cues simultaneously when making JOLs (see also Soderstrom & McCabe, 2011), though under some conditions, certain cues might be relied upon more heavily than others (Pachur & Bröder, 2013). Koriat’s cue-utilization theory mainly focuses on JOLs for oneself, but it provides clues for how people might make JOLs for others and why it may be difficult to do so. More specifically, individuals may be able to predict others’ memory performance by using commonly shared intrinsic and extrinsic cues, but a lack of access to another person’s mnemonic cues makes predictions for others a challenging task. Koriat claimed that if individuals gain access to others’ mnemonic cues, they should be able to predict learners’ performance accurately. We now review the extant literature on making JOLs for others.

## JOLs for others

### Definition and measures of accuracy

JOLs for others refer to how individuals judge others’ learning or how individuals predict others’ future performance. Similar to JOLs made for oneself, JOLs for others can be made on an item-by-item basis or in aggregate form. Soderstrom et al. (2016) claimed that it is often imperative to be able to accurately estimate others’ learning, such as when teachers assess their students’ comprehension, but in comparison to a substantial body of research on JOLs for oneself, there is relatively little research on JOLs for others, although there is research in other domains that is tangentially relevant to this topic. Examples include social psychology and language (SPL) studies that focus on language production in social scenarios (Smith, 1983); studies on attribution theory that investigate how individuals explain the causes of another person’s behavior (for a review, see Harvey & Weary, 1984); and knowledge estimation studies that inquire about how people estimate someone else’s level of understanding of particular topics (Thomas & Jacoby, 2013). Although these research areas seek to explain how people make judgments about others, to our knowledge, relatively few studies have probed specifically into JOLs for others.

Similar to JOLs for oneself, the accuracy of making JOLs for others can be measured via calibration (absolute accuracy) and resolution (relative accuracy; for a review, see Thiede et al., 2019). In this context, calibration refers to the absolute difference between judges’ JOLs and learners’ performance. For example, suppose a teacher predicts a student will score a 90/100 on an upcoming test but the student actually scores an 80/100. In this case, the teacher was 10% over-confident, which is an index of absolute accuracy. Resolution, on the other hand, refers to the finer-grained correspondence between judges’ JOLs and learners’ performance. If, for example, the teacher’s JOLs differentiate between those students who performed well on the test versus those who performed poorly, resolution would be relatively high. Several studies have reported absolute accuracy when reporting JOL for others; however, most studies in this domain have focused on resolution, as this has been the recommended index of metacognitive accuracy (Nelson, 1984).

In the following sections, we review both laboratory- and classroom-based studies concerning making JOLs for others. In doing so, we seek to highlight the functions, mechanisms, and accuracy of such judgments.

### Functions of JOLs for others

Similar to previous findings that JOLs can help individuals regulate their subsequent study sessions and that accurate JOLs lead to more effective learning (e.g., Thiede et al., 2003), individuals should be able to better regulate their learning after receiving accurate judgments from others. In addition, making accurate JOLs for others helps individuals better understand how to instruct others. For example, when teachers make accurate JOLs for students, they can adjust their instruction based on students’ learning, ultimately leading to better performance among students. Thiede et al. (2015) found that the accuracy of teachers’ JOLs is positively related to students’ academic performance through effective instruction. Further, monitoring and feedback via formative assessments are positively related to the relative accuracy of JOLs for students, and formative assessments are encouraged in classrooms to improve students’ learning outcomes. (see Thiede et al., 2019,. for more information). Thus, accurate JOLs for others can benefit learning by enhancing instruction and improving subsequent self-regulated learning strategies.

### Mechanisms for JOLs for others

There is no comprehensive model for JOLs for others. As a result, we start with Koriat’s (1997) cue-utilization model and then review extant articles on JOLs for others, categorizing these findings to highlight the connections between the empirical evidence and cue-utilization theory. Also, we compare those findings to cue-utilization theory to illustrate what factors are needed to update the cue-utilization model from a JOLs-for-oneself framework to one applicable to JOLs for others. Our aim in this section is to review articles that examined how individuals use theory- and experience-based cues when making JOLs for others. We note, however, that for most of these studies, making JOLs for others was not the primary focus. Nonetheless, these studies are informative for our purposes.

#### JOLs for others in laboratory-based research

##### Theory-based cues can be utilized to make JOLs for others

Tullis and Fraundorf (2017) demonstrated that individuals can make JOLs for others based on theory-based intrinsic cues – in this case, the associative strength between cues and target words – even though the judge does not have learning experiences related to those cues. In their Experiment 1, participants were randomly assigned to either a generation condition or a learning condition. Those in the generation condition were instructed to read words and generate a memory cue for each word, whereas participants in the learning condition learned the cue-word association. In Experiment 2, they further divided the generation group into a generator and an observer group, where participants in the observer group observed cues generated by generators and predicted learners’ future performance. Across both experiments, the accuracy of JOLs for learners made by generators and observers was higher than chance but lower than JOLs made by learners for themselves. There was no difference in the JOL accuracy between generators and observers, and associative strength was the most relied upon cue for making JOLs. This suggests that, in the absence of their own mnemonic cues, individuals rely on theory-based analytic cues (e.g., associative strength) to make JOLs for others. Furthermore, the finding that JOLs for oneself were more accurate than JOLs for others suggests that additional mnemonic cues were diagnostic of one’s own later performance and, therefore, add to the accuracy of those JOLs.

Koriat et al. (2004) found that individuals take retention intervals (extrinsic cues) into consideration when making JOLs for others but only when the retention interval is manipulated within subjects. In their study, participants studied word lists and predicted their future performance for three different retention intervals (10 min/1 day/1 week). Participants showed a stability bias in their JOLs when the different retention intervals were manipulated between participants. Specifically, participants predicted similar levels of performance no matter what the retention interval was. Similarly, when the experimenters asked other participants to predict the learners’ performance, JOLs for others were nearly identical across three retention intervals. However, the pattern was different for both JOLs for oneself and JOLs for others when retention interval was manipulated in a within-participants manner because, in that case, the retention interval cue was made salient and thus participants used that information to inform their JOLs (i.e., lower JOLs were given for longer retention intervals). These findings suggest that when individuals do not have their own mnemonic cues, they rely on theory-based extrinsic cues (in this case, retention intervals) to inform their JOLs for others, but this effect might be detected only when a within-subjects design is used.

Also, Mueller et al. (2014) found a significant main effect of font size such that individuals believed a larger font size would lead to better memory in others. In their Experiments 3a and 3b, participants were instructed to read the scenario of learners studying word lists in small and large font sizes, and they found that participants believed that words in larger font size would produce relatively greater memory gains in others (see also Kornell et al., 2011). Thus, font size is also an intrinsic theory-based cue that people can utilize to make JOLs for other learners.

Finally, Undorf and Erdfelder (2013) found that judges use study time to predict learners’ future performance. In their three experiments, they told judges true or false study times of learners on each item and asked judges to make JOLs for learners. The relative accuracy between judges’ JOLs for learners and learners’ test performance was different between true and false study-time conditions, suggesting that individuals utilize learners’ study time as a theory-based extrinsic cue to make JOLs for others.

These findings suggest that theory-based cues, regardless of whether they are intrinsic or extrinsic, can inform JOLs for others, which is consistent with Koriat’s (1997) cue-utilization theory. Furthermore, Kelley and Jacoby (1996) pointed out that a lack of subjective learning experience leads to a reliance on theory-based heuristics when making JOLs for others. That is, when individuals have experiences learning something, they can make use of their own learning experiences to predict others’ performance. However, when they do not have their own mnemonic cues to rely upon, they tend to use theory-based cues when making JOLs for others.

##### JOLs for others are relatively accurate when mnemonic cues are available

Vesonder and Voss (1985), used the Learner–Listener–Observer (LLO) paradigm to show that individuals can make accurate JOLs for others when mnemonic cues are available. In this paradigm, Learners studied declarative sentences, made item-by-item predictions after each sentence, and then completed a final recall test. Listeners and Observers also read the sentences and predicted the Learners’ performance, but during the recall phase, Listeners heard the recall responses of Learners whereas the Observers did not (they heard a noise instead). This prediction-recall cycle was repeated several times. Results of the first cycle revealed no significant difference in prediction accuracy among the three groups, with participants in all conditions overestimating the Learners’ performance. This is not surprising given the only cues available to inform JOLs during the first cycle were related to the materials themselves (intrinsic cues). However, on subsequent cycles, Learners and Listeners showed higher prediction accuracy than Observers because they acquired mnemonic cues relevant to the Learners’ performance (there was no significant difference in JOLs accuracy between Learners and Listeners). These findings support the cue-utilization theory by showing that: (1) in the absence of other information, intrinsic cues are often used to inform JOLs, and (2) when people have access to others’ mnemonic cues, the accuracy of JOLs for other learners can be improved.

Similarly, Serra and Ariel (2014) claimed that past performance of learners can help judges make accurate JOLs for other learners. They divided their participants into Learners and Observers. Learners studied word pairs and then attempted to retrieve the items on a later test. Both Learners and Observers were informed of the performance of Learners on trial 1 and were then instructed to predict Learners’ performance on trial 2. Past test performance helped both groups distinguish between retrievable and non-retrievable items and thus improved subsequent JOL accuracy. However, Learners can better use their past performance than observers to make more accurate JOLs. This is because, although prior performance of Learners was made available to Observers, Learners likely relied on other idiosyncratic experiences that Observers did not have access to. Consistent with the cue-utilization theory, these results provide further evidence that mnemonic cues are diagnostic of future performance and, if others have access to those cues, accuracy of JOL for others can be improved.

Matvey et al. (2001) found that generation latency of learners, a mnemonic cue, can be utilized by others to predict learners’ generation task performance. In their study, participants were randomly assigned to three groups: learner, observer, and judge. Participants in the learner group generated target words based on cues and task requirements, and their generation latency (time between presentation of cues and generating target words) was recorded. Observers were yoked to learners and read the materials, together with the generation latency of learners. Judges were also yoked to learners, but they only read the learned materials without knowing the generation latency of learners. Then, participants in all three groups made JOLs for learners to predict learners’ test performance. For learners and observers (who both had access to the learner’s mnemonic cues), generation latency was negatively related to JOLs for others, such that longer latencies were associated with lower JOLs. These results suggest that, when available, individuals can utilize others’ mnemonic cues like generation latency to predict others’ performance.

The empirical findings summarized thus far suggest that a major impediment to making accurate JOLs for others is that individuals do not have access to others’ mnemonic information. However, when mnemonic information *is* available, individuals have a propensity to utilize such cues (e.g., past performance) to predict others’ future performance. For instance, Helzer and Dunning (2012) found that individuals think past performance is of greater significance compared to aspirational cues. Children also make JOLs for others based on performance cues, whereas they make JOLs for themselves based on their own wishful thinking. As a result, children’s JOLs for others tend to be more accurate than their JOLs for themselves (e.g., Lipko et al., 2009; Schneider, 1998; Stipek et al., 1984). If individuals have more diagnostic mnemonic cues about others’ memory (e.g., generation latency; Matvey et al., 2001), they should be able to make as accurate JOLs for others as learners do for themselves (Koriat, 1997; Vesonder & Voss, 1985). It is worth noting that learners’ mnemonic cues function as theory-based analytical cues when judged by others, which means judges can predict learners’ performance based on these cues without access to learners’ subjective experiences.

##### Individuals might interpret cues differently according to their prior learning experiences

A study by Koriat and Ackerman (2010) demonstrated that individuals may interpret cues in a different manner depending on their own learning experiences. They conducted three experiments to examine cue-utilization for both themselves and others. In their experiments, they included a Self-Learning condition and an Other-Learning condition. Participants in the Self condition were instructed to study word lists in a self-paced manner and make JOLs for themselves after studying the words. Participants in the Other condition watched a video showing the self-paced study process of another student and made JOLs for the student in the video. The experimenters manipulated the order of the study phase and the study time of the student in the video. Specifically, some participants studied the word lists themselves first and watched the video second, whereas others watched the video first and studied the word lists second. Some items in the video were presented to the student for 5 s, and other items were presented for 10 s. It was shown that individuals predicted an inverse relationship between study time and learning performance when making JOLs for *themselves*. That is, they interpreted needing to spend relatively more time on some items as a clue that those items were harder to learn and, therefore, gave lower JOLs for those items. However, it was also revealed that individuals assume the opposite relationship between study time and difficulty – namely, that devoting more time to an item will produce *better* memory – when making JOLs for *others*. Furthermore, the disparity in how study time is used to inform JOLs for oneself versus others depended on the order of those conditions. When participants learned word lists themselves first, they used their own learning experience to subsequently judge others’ learning, but if they watched the video first, they used an analytical inference (i.e., more time spent on items should result in better memory) to judge others’ learning. Therefore, the authors argued that when judging learning for self versus others, individuals might interpret the effects of extrinsic cues differently, and some extrinsic cues (study time in this case) may influence JOLs for others through a theory-based analytic process or an experience-based heuristic process, depending on whether the judge had the same or similar learning experiences.

Undorf and Erdfelder (2011) used the same paradigm to demonstrate that judges’ prior learning experiences affect how they make JOLs for others when study time is known. In their study, all participants were instructed to complete learning and observing tasks. For the learning task, participants learned word pairs, made JOLs for themselves, and completed a later memory test in a self-paced manner. For the observing task, participants observed the word pairs and were informed of the study time of a random participant in the leaning condition for each item. Then, participants made JOLs for that learner. The researchers manipulated the order of the tasks by asking some participants to complete the learning task first and asking others to complete the observing task first. An interaction was found such that if participants learned the word pairs first, the correlation between their study time and JOLs for others was negative, whereas if participants observed the word pairs first, the correlation between study time and JOLs for others was positive. Therefore, this study indicated that previous learning experiences of judges can influence how they interpret theory-based cues and, in turn, how they make JOLs for others.

##### Predictions are inaccurate when inadequate cues are provided

Tirso and Geraci (2020) measured participants’ absolute and relative JOL accuracy for others in a wide variety of situations, including in classroom and laboratory contexts, when judges did not know much about the learners. Their findings indicated that individuals tend to be over-confident about others’ performance compared to their own performance unless they perceive the other person as unlikeable, in which case predictions for others are much lower than self-predictions. Tirso and Geraci proposed an information-motivation theory, stating that if individuals have insufficient cues for others’ learning, they are inclined to overestimate others’ performance with the motivation to view others in a positive way.

Miller and Geraci (2016) provided additional evidence that judges tend to overestimate learners’ performance when they do not have sufficient information about the learner. In their study, Learners studied paired-associates and predicted their performance (original JOLs). After making original JOLs, Learners were prompted to complete a retrieval practice task in which they tried to recall 10% of the previously studied paired associates, and they were then to adjust their original JOLs (adjusted JOLs) after retrieval practice. Judges read the original JOLs and retrieval performance of the Learners they were yoked to and then made adjusted JOLs for learners. The results showed that Learners made lower and more accurate JOLs after retrieval practice and that Judges made inflated and inaccurate adjusted JOLs for learners. These findings indicate that (1) partial retrieval practice is a subjective and idiosyncratic cue that others cannot utilize, and (2) individuals tend to be over-confident about others’ performance when insufficient cues are available.

Importantly, however, inadequate cues do not always lead judges to overestimate the learner; sometimes there are so few cues that judges’ JOLs of learners are at chance level. As an example, Helm and Growns (2023) reported low accuracy of JOLs for others on a face-recognition test when judges had insufficient cues. In their Experiment 1, participants were instructed to observe different face pictures and predict their own performance and that of others by selecting “Yes” or “No” for each picture. Their prediction was categorized as correct if they selected “yes” when the yoked participant recognized the face picture correctly, or if they selected “no” when the yoked participant did not recognize the face picture. The results suggested that JOLs for oneself are more accurate than JOLs for others, and that JOLs for others are not more accurate than chance (50%). In their Experiment 2, the experimenters manipulated the presentation time and size of the pictures to detect the influence of various factors on JOLs for oneself and others. Helm and Growns found that individuals make higher JOLs for both self and others for larger faces compared to smaller faces, but those JOLs are not influenced by presentation time. Therefore, the study of Helm and Growns demonstrated that when individuals do not have access to either diagnostic intrinsic cues (associative strength, emotionality, etc.) or mnemonic cues, their JOLs for others are not more accurate than chance.

#### JOLS for others in the classroom

Teachers can be engaged in many cognitive and metacognitive activities in classrooms (for a review, see Duffy et al., 2009). One task that is particularly important for teachers to carry out is to judge their students’ learning accurately in order to adjust their instruction effectively and efficiently. We turn next to a discussion of how teachers make JOLs for their students, because these findings demonstrate how individuals judge others’ learning in a more realistic setting and thus highlight information that should be considered when generating a model for how JOLs are made for others.

##### Teachers rely heavily on performance cues and take social cues into consideration

Though some studies have demonstrated that the accuracy of teachers’ JOLs for students is relatively low (e.g., Engelen et al., 2018), several meta-analyses have shown that the accuracy of JOLs for students tend to be relatively high (Hoge & Coladarci, 1989; Kaufmann, 2020; Südkamp et al., 2012). This high accuracy of teachers’ JOLs for students can be attributed to the fact that teachers often ground their judgments in students’ previous test performance (Hecht & Greenfield, 2002; Martínez et al., 2009). Consistent with the findings of Koriat (1997), mnemonic cues (like previous performance) are highly diagnostic of future performance, and if mnemonic cues can be discerned by others, others should be able to predict individuals’ performance accurately. It is worth noting that the diagnosticity of prior test performance also depends on the alignment between previous and future tasks, and more alignment leads to better accuracy (Thiede et al., 2019).

In a meta-analysis by Südkamp et al. (2012), the authors proposed a model to illustrate the cues that can influence teachers’ JOLs for students. Specifically, teachers’ JOLs are based on four types of cues: intrinsic cues (e.g., test characteristics), extrinsic cues (e.g., multiple tests vs. only one test), mnemonic cues (e.g., prior performance of students), and social cues – cues that are not directly relevant to learning experiences but could nonetheless be predictive of students’ future performance (e.g., age, personality traits, mood, body language, etc.). The utilization of these cues can influence the accuracy of prospective judgments made by teachers regarding their students’ learning. Resembling the findings that individuals can utilize multiple intrinsic and extrinsic cues at the same time (Undorf et al., 2018), teachers consider many cues when making JOLs in classrooms. van de Pol et al. (2021a) revealed that teachers used, on average, 5.87 cues when making JOLs for students, with performance cues being used most frequently, followed by student cues and task cues. Their findings also suggest that it is sometimes difficult for teachers to interpret and judge the diagnostic value of a cue. In another study, van de Pol et al. (2021b) found that performance cues are more diagnostic than other cues. According to this research, teachers utilize 6.35 cues on average, most of which are highly diagnostic cues, although teachers tend to overestimate cue value. Some researchers have claimed that providing only diagnostic cues for teachers will enhance the accuracy of their JOLs for students (e.g., Oudman et al., 2018; cf. van de Pol et al., 2021a). Thus, in real classroom settings, teachers base their JOLs on multiple cues, but they tend to use the most diagnostic ones – performance cues (Griffin et al., 2009; Thiede et al., 2010; van de Pol et al., 2019).

Despite the relatively high accuracy of teachers’ JOLs for students, variation in accuracy is large (Südkamp et al., 2012), and this is because teachers’ JOLs for students are susceptible to many misleading factors (Meissel et al., 2017). For example, some studies show that teachers’ judgments are affected by gender (e.g., Helwig et al., 2001; Mizala et al., 2015) and the ethnicity of students (Martínez et al., 2009), although other studies have shown that demographic information only accounts for a very small proportion (~ 2%) of variation in their judgments (e.g., Kaiser et al., 2017). Further, teachers’ JOLs for students are vulnerable to additional misleading cues, including student engagement (e.g., Jenkins & Demaray, 2015; Kaiser et al., 2013), interest (e.g., Kikas et al., 2015), behaviors (e.g., Bennett et al., 1993), and so on (for more details, see Thiede et al., 2019). These cues are not necessarily diagnostic of students’ future performance, but teachers might misinterpret them as diagnostic cues. In addition, when making JOLs for students, teachers usually take students’ backgrounds into consideration, such as their personality (e.g., Rausch et al., 2015) and how much their parents value education (e.g., Hauser-Cram et al., 2003). In a recent review by Costa et al. (2024), background information that influences students’ academic achievements is categorized into two groups: (1) personal factors such as substance use, etc. and (2) contextual factors such as family, school, and peer environment factors. Thus, background information may or may not be diagnostic of students’ learning performance, but if teachers believe in low-diagnostic cues, their JOLs for students will be biased. These findings suggest that individuals are likely to take *social cues* into consideration when making JOLs for others. Therefore, compared to making JOLs for oneself, making accurate JOLs for others is likely more difficult because a multitude of misleading social cues may influence individuals’ judgments of others.

##### Artificial intelligence (AI) uses cues that may or may not be directly related to learning to predict learners’ performance

Azevedo (2007) claimed that one function of advanced learning technology is to assist students in their metacognitive processes, including judgments of learning and self-regulated learning. MetaTutor, as an example of advanced learning technology, utilizes many cues to predict learners’ performance and adjusts its learning parameters to help learners achieve better test performance. These cues not only include learning-relevant cues such as study time, frequency of studying, test performance, and feedback, but also encompass cues that are not directly related to learning but can reflect learners’ mental states, such as emotion via facial recognition, eye movements, and heart rate variability (e.g., Dever et al., 2025; Sobocinski et al., 2024; for reviews, see Azevedo et al., 2011, 2022; Azevedo & Dever, 2021; Azevedo & Taub, 2020). Therefore, a variety of cues (either directly related to learning or not) are used by AI to predict learners’ future performance.

#### Summary of existing studies on making JOLs for others

Making JOLs for others is an inferential process that depends on many cues. Specifically, individuals can leverage theory-based intrinsic and extrinsic cues to predict others’ performance (e.g., Tullis & Fraundorf, 2017), and if they have access to others’ idiosyncratic mnemonic cues, individuals are able to make highly accurate JOLs for others (e.g., Matvey et al., 2001; Vesonder & Voss, 1985). However, not all intrinsic and extrinsic cues are purely theory-based, analytic cues. Some intrinsic and extrinsic can also influence JOLs for others through mnemonic cues so long as judges experience the cues and are then given an opportunity to predict others’ performance after such experiences (Undorf & Erdfelder, 2013). This explains why individuals interpret study time in different ways when making JOLs for oneself versus others if they did not learn the materials themselves before making the JOLs (Koriat & Ackerman, 2010; Undorf & Erdfelder, 2011). It is worth noting that although individuals often make JOLs for others based on their own learning experiences, there are limitations in terms of how those experiences are applicable to others (Laursen & Fiacconi, 2022). When individuals do not have enough cues to predict others’ performance, they are inclined to overestimate others’ performance (e.g., Miller & Geraci, 2016; Tirso & Geraci, 2020) or provide JOLs that are not more accurate than chance (e.g., Helm & Growns, 2023). On one hand, these findings align with Koriat’s (1997) cue-utilization theory in that individuals can utilize theory-based cues when making JOLs for others, and when they have access to mnemonic cues of learners, they can make relatively accurate JOLs for others. On the other hand, the extant literature demonstrates that people often use additional cues that are not captured in Koriat’s model. For instance, JOLs for others can be influenced by the judges’ subjective experiences (e.g., Kelley & Jacoby, 1996; Serra & Ariel, 2014; Undorf & Erdfelder, 2011, 2013); sometimes individuals judge others’ knowledge in accordance with their own knowledge, which is termed the curse-of-knowledge effect (e.g., Camerer et al., 1989; for a review, see Nickerson, 1999). Therefore, extant findings on JOLs for others demonstrate that the cue-utilization theory is a useful model when predicting others’ performance, but more information (e.g., judge’s experience) needs to be incorporated into the JOLs-for-others model. In addition, the findings related to how teachers and AI make JOLs for students support and extend Koriat’s cue-utilization theory to a large extent. First, teachers and AI predict students’ performance based on intrinsic, extrinsic, and mnemonic cues. Second, mnemonic cues (past performance) are most diagnostic, and if individuals have access to learners’ mnemonic cues, they can make accurate JOLs for others. However, given that JOLs made by teachers and AI are also influenced by social cues, it seems worthwhile to update Koriat’s model such that these cues are included when discussing how JOL’s are made for others.

## Cue-utilization model of JOLs for others

**Fig. 2:**
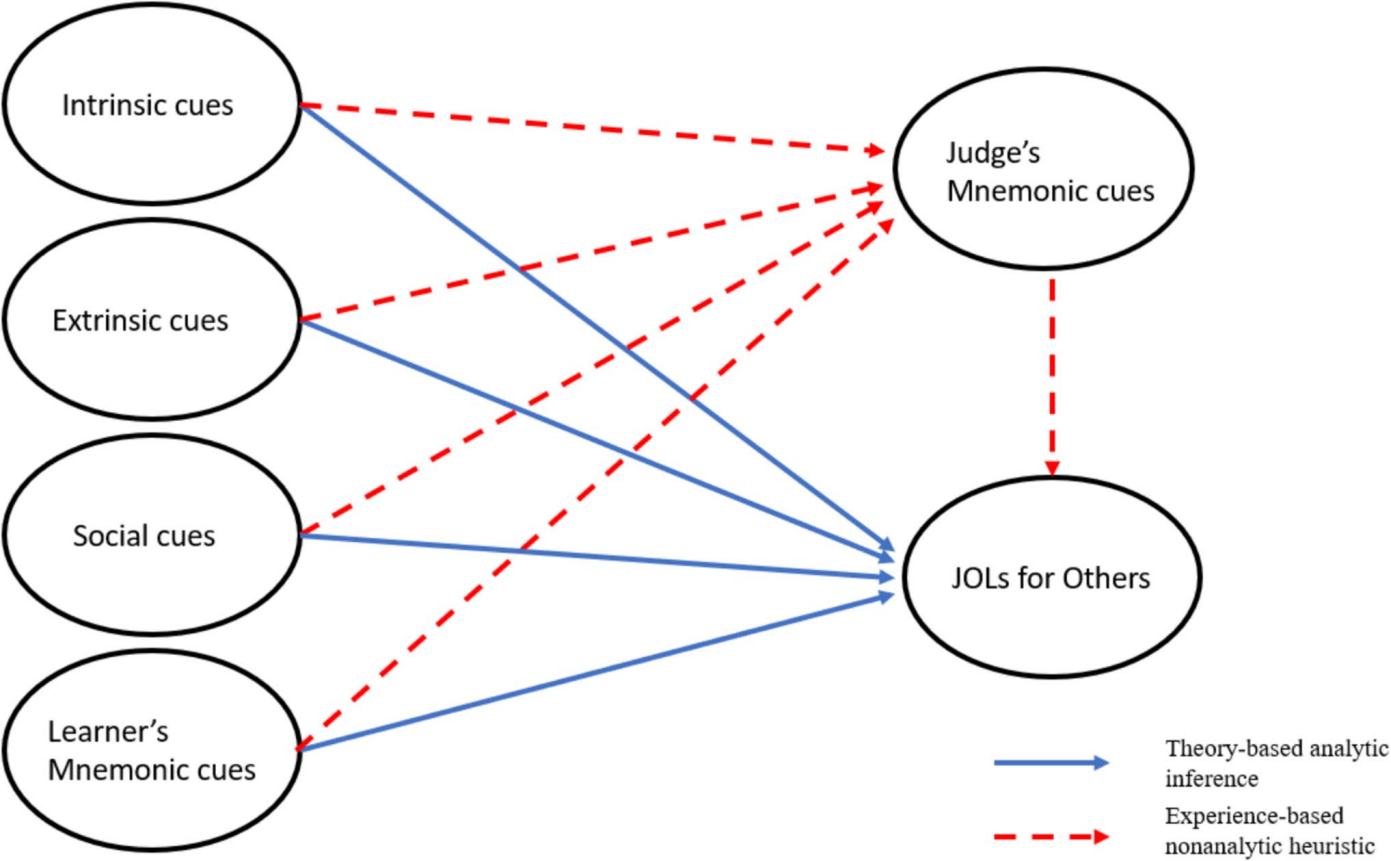
A cue-utilization model for making judgments of learning (JOLs) for others

Koriat (1997) proposed a cue-utilization theory to illustrate how intrinsic, extrinsic, and mnemonic cues are used when people make JOLs for themselves. This model, however, is not entirely suitable for describing how JOLs are made for others because: (1) when individuals make JOLs for others, their JOLs are influenced by contextual cues not captured in Koriat’s model; (2) cues can be divided into theory-based cues and experience-based cues, where theory-based cues can be used by others, but experience-based cues can only be used by the learners themselves; and (3) judges’ relevant learning experiences may influence their JOLs for other learners. As a result, an updated model is necessary to better delineate how JOLs are made for others. Our new model is shown in Fig. 2. It retains the major elements of Koriat’s cue-utilization model but also includes information relevant to making JOLs for others.

### Cues in the model

Some cues that are used to make JOLs for others are included in Koriat’s (1997) cue-utilization model. Specifically, when making JOLs for others, individuals are able to utilize intrinsic cues – characteristics of learned materials – to predict others’ performance, such as associative strength (Tullis & Fraundorf, 2017) and font size (Mueller et al., 2014). Also, consistent with Koriat, extrinsic cues – learning conditions and strategies – can be used by judges to make JOLs for learners, including retention interval (Koriat et al., 2004) and study time (Koriat & Ackerman, 2010). As a result, intrinsic and extrinsic cues should be included in the JOLs-for-others model. The mnemonic cues from Koriat’s model (here depicted as Judge’s mnemonic cues) should also be included in this new model because judges’ subjective learning experiences influence how they interpret various cues (e.g., Kelley & Jacoby, 1996; Koriat & Ackerman, 2010; Nickerson, 1999). It is worth noting that, similar to Koriat’s model, intrinsic and extrinsic cues influence JOLs for others directly through theory-based analytical process or indirectly through judges’ experience-based heuristics.

Importantly, the new model includes another category of cues that individuals utilize to judge others’ learning which are not captured in Koriat’s (1997) model. Specifically, when making JOLs for others, judges are influenced by social cues that are not directly relevant to the learning itself (these cues are not considered when learners make JOLs for themselves). For example, the likeability of learners will influence how judges make JOLs for them (Tirso & Geraci, 2020), and teachers take students’ background information into consideration when making predictions (Thiede et al., 2019). Furthermore, learners’ mnemonic cues, such as generation latency (Matvey et al., 2001) and past performance (e.g., Serra & Ariel, 2014; Vesonder & Voss, 1985), should be included in the model because judges can apply their theories regarding these mnemonic cues to predict learners’ performance. Importantly, social cues and learner’s mnemonic cues can affect JOLs for others in both direct (belief-based) and indirect (through judge’s mnemonic cues) ways. For example, if a judge predicts the performance of two students, and he/she knows that student A has a part-time job (social cue), but student B does not, then the judge may think student B will perform better because this student has more time for studying. However, if the judge had a part-time job in the past but still excelled in the class, then the judge might take his/her own experiences into consideration and conclude that this social cue should be discounted when judging others’ learning. Likewise, past performance (learner’s mnemonic cue) is believed to be a good indicator of future performance by others (e.g., Helzer & Dunning, 2012), and access to this mnemonic cue can directly influence JOLs for others. However, past performance can also influence JOLs for others indirectly through the *judge’s* mnemonic cues. For example, if the judge performed well on the first exam in the class but performed poorly on the second one, the judge may realize that the correspondence between past and current performance might not be very strong, leading him/her to discount the effect of past performance.

Therefore, in our new model, we not only include cues from Koriat’s model to show how intrinsic, extrinsic, and mnemonic cues can affect JOLs for others, but we also incorporate social cues and learner’s mnemonic cues that may affect JOLs for others and delineate the relationships between different cues and JOLs.

### Comparison between the two models

Our JOLs-for-others model is similar to Koriat’s (1997) cue-utilization model in some aspects because: (1) we believe that making JOLs for others is an inferential process based on various cues (e.g., intrinsic, extrinsic, and mnemonic cues); and (2) some cues are commonly shared theory-based cues that can be utilized by others, whereas some cues are experience-based and can only be utilized by learners. Nevertheless, our model is different from Koriat’s model in significant ways. First, social cues are included in our model because it is clear that such cues are considered when making JOLs for others (for a review, see Thiede et al., 2019). Second, mnemonic cues are divided into learner’s mnemonic cues and judge’s mnemonic cues. Learner’s mnemonic cues (e.g., past performance, generation latency, etc.) refer to theory-based cues that, if known, judges can use directly when predicting the learning of others (e.g., Serra & Ariel, 2014; Vesonder & Voss, 1985). Judge’s mnemonic cues are based on the judge’s own experiences (i.e., they are experienced-based) and can also be utilized to predict others’ performance (e.g., the curse of knowledge effect; Nickerson, 1999); Finally, judge’s mnemonic cues are a mediator between theory-based cues and JOLs for others because a judge’s past experiences may influence how he/she interprets theory-based cues (Koriat & Ackerman, 2010; Undorf & Erdfelder, 2011, 2013). As previously mentioned, individuals interpret the relationship between others’ study time and their future performance in a different way depending on whether the judge has experienced the learning situation himself/herself.

### Future directions

Individuals can make JOLs based on various cues (intrinsic, extrinsic, social, and mnemonic) for oneself and others, but it is more challenging to predict others’ performance because we rarely have access to the more diagnostic, mnemonic cues that would help us make accurate predictions. Further, making JOLs for others is complicated because various conditions provide individuals with different types of cues and different numbers of cues. When individuals have access to many cues, it is unknown how people will make JOLs for others by prioritizing different cues in a social context. Thus, future research should examine the relationships among various cues and how individuals combine different cues to make JOLs for others.

In previous JOLs-for-others studies, experimenters manipulated the cues that judges have access to with the aim of understanding the experience-based or theory-based characteristics of the cues (e.g., Koriat et al., 2004; Matvey et al., 2001; Serra & Ariel, 2014). In such cases, participants have no other available cues to serve as the basis for making JOLs for others. However, in our daily lives, individuals have access to a myriad of cues. For example, teachers have access to various cues (Südkamp et al., 2012). Students also have many cues available about their peers, such as motivation, past performance, background, etc. Although individuals take many cues into consideration when making JOLs for themselves (Undorf et al., 2018) and teachers utilize, on average, more than five cues when predicting student learning in classrooms (van de Pol et al., 2021a, 2021b), it is still largely unknown how individuals predict others’ performance in a cue-abundant environment. Specifically, it is worth investigating how individuals value and prioritize various cues when making JOLs for others and how prioritizing various cues influences the accuracy of JOLs for others. Furthermore, future studies should investigate which cues are more diagnostic than others, especially given that JOLs for others are influenced by both the judge’s idiosyncratic experiences and commonly shared theory-based cues. Research utilizing structural equation modeling may be particularly well suited to delve into these complex issues.

## Conclusion

Similar to making JOLs for oneself, making JOLs for others is an inferential process based on many different cues. As our new model indicates, however, judging others’ performance is particularly challenging because, compared to JOLs for oneself, such judgments are susceptible to the influence of more cues, particularly judges’ mnemonic cues and social cues. Given that our behaviors are directly linked to our beliefs, it is imperative to probe the cues that individuals prioritize when making JOLs for others and to explore how the accuracy of these judgments can be improved.

## Data Availability

Not applicable.

## Declarations

### Open Practices Statement

No data or materials for this review are available, and this review is not pre-registered.
